# Learning locality-sensitive bucketing functions

**DOI:** 10.1093/bioinformatics/btae228

**Published:** 2024-06-28

**Authors:** Xin Yuan, Ke Chen, Xiang Li, Qian Shi, Mingfu Shao

**Affiliations:** Department of Computer Science and Engineering, The Pennsylvania State University, University Park, PA16802, United States; Department of Computer Science and Engineering, The Pennsylvania State University, University Park, PA16802, United States; Department of Computer Science and Engineering, The Pennsylvania State University, University Park, PA16802, United States; Department of Computer Science and Engineering, The Pennsylvania State University, University Park, PA16802, United States; Department of Computer Science and Engineering, The Pennsylvania State University, University Park, PA16802, United States; Huck Institutes of the Life Sciences, The Pennsylvania State University, University Park, PA 16802, United States

## Abstract

**Motivation:**

Many tasks in sequence analysis ask to identify biologically related sequences in a large set. The edit distance, being a sensible model for both evolution and sequencing error, is widely used in these tasks as a measure. The resulting computational problem—to recognize all pairs of sequences within a small edit distance—turns out to be exceedingly difficult, since the edit distance is known to be notoriously expensive to compute and that all-versus-all comparison is simply not acceptable with millions or billions of sequences. Among many attempts, we recently proposed the locality-sensitive bucketing (LSB) functions to meet this challenge. Formally, a $\left(d_{1},d_{2}\right)$-LSB function sends sequences into multiple buckets with the guarantee that pairs of sequences of edit distance at most $d_{1}$ can be found within a same bucket while those of edit distance at least $d_{2}$ do not share any. LSB functions generalize the locality-sensitive hashing (LSH) functions and admit favorable properties, with a notable highlight being that optimal LSB functions for certain $\left(d_{1},d_{2}\right)$ exist. LSB functions hold the potential of solving above problems optimally, but the existence of LSB functions for more general $\left(d_{1},d_{2}\right)$ remains unclear, let alone constructing them for practical use.

**Results:**

In this work, we aim to utilize machine learning techniques to train LSB functions. With the development of a novel loss function and insights in the neural network structures that can potentially extend beyond this specific task, we obtained LSB functions that exhibit nearly perfect accuracy for certain $\left(d_{1},d_{2}\right)$, matching our theoretical results, and high accuracy for many others. Comparing to the state-of-the-art LSH method Order Min Hash, the trained LSB functions achieve a 2- to 5-fold improvement on the sensitivity of recognizing similar sequences. An experiment on analyzing erroneous cell barcode data is also included to demonstrate the application of the trained LSB functions.

**Availability and implementation:**

The code for the training process and the structure of trained models are freely available at https://github.com/Shao-Group/lsb-learn.

## 1 Introduction

The most time-consuming computational task behind many instances of large-scale sequence comparison, such as the construction of overlap graphs, phylogeny reconstruction, and (multiple) sequence alignment, can be described as identifying all pairs of similar sequences in a given set or across multiple sets. Scalable algorithms are still missing for this fundamental problem. The challenge is twofold. First, many applications require the comparison of divergent sequences or data with high-error rate, but the edit distance, a basic, commonly used model for evolution, is not easy to work with. For example, the edit distance between two sequences is likely not computable in strongly subquadratic time (Backurs and Indyk 2018). One line of research tries to cope with this difficulty by embedding the space of sequences equipped with edit distance into other more well-studied metric spaces, such as real vectors with $\ell^{1}$-norm (Ostrovsky and Rabani 2007), $\ell^{2}$-norm (Dai *et al.* 2020), or sequences with Hamming distance (Chakraborty *et al.* 2016). Such an embedding f maps arbitrary sequences x and y into elements f(x) and f(y) of the new space satisfying edit(x,y) approximates d(f(x),f(y)) where edit(·,·) is the edit distance and d(·,·) is the metric of the new space which can be computed with a lower time complexity, often in linear or even sublinear time. Unfortunately, the ease of computing of the embedded metric comes at the cost of accuracy, namely, the embedded distances can be distorted. Designing low distortion embeddings for the edit distance appears to be hard and is sometimes proved to be impossible (Krauthgamer and Rabani 2009). Furthermore, an easier-to-compute metric by itself does not fully solve the large-scale sequence comparison problem due to the second difficulty: the amount of sequences to be compared is enormous, usually in the range of millions, if not billions, which renders the direct pairwise comparison infeasible regardless of the metric used.

The key observation towards addressing the second challenge is that in real applications, the majority of the comparisons do not produce positive results and are thus wasteful. In other words, the total number of interested pairs, for example, all the overlapping pairs of reads, is usually linear, rather than quadratic, with respect to the size of the input. To take advantage of this observation, bucketing is used to quickly rule out irrelevant pairs. Sequences that share some common features, such as kmers (Broder 1997), subsequences (Li *et al.* 2023), or their hash values, are grouped into the same bucket; then pairwise comparisons are only applied among sequences within the buckets. To achieve a balance between sensitivity and computation time, the bucketing features are expected to roughly capture the similarity of the sequences that they represent. Locality-sensitive hashing (LSH) formalizes this idea. A family of hash functions is $\left(d_{1},d_{2}\right)$-sensitive for a distance function d(·,·) if the probability that two sequences x and y have a hash collision (i.e., they are assigned to the same bucket) is high when $d(x,y)\;\leq \;d_{1}$, and the probability is low when $d(x,y)\;\geq \;d_{2}$. An LSH function is called ungapped if $d_{1}=d_{2}\;-\;1$ where a guarantee on the probability of hash collision exists for all distance values; it is gapped if $d_{1}\;<\;d_{2}\;-\;1$ where the function poses no requirement for distances in the range of $\left[d_{1}\;+\;1,d_{2}\;-\;1\right]$. LSH functions for the normed metrics such as the Jaccard distance (Broder 1997) and the Hamming distance exist, but designing LSH functions for the edit distance is exceedingly difficult. A simple reasoning can show that perfect, deterministic LSH functions do not exist: if there is such a function f which satisfies f(x)=f(y) with probability 1 for every pair of x and y with $\text{edit}(x,y)\;\leq \;d_{1}$, then for any pair x and z with $\text{edit}(x,z)\;\geq \;d_{2}$ one can always find a series of sequences $y_{1},y_{2},\ldots ,y_{t}$ such that $\text{edit}(x,y_{1})=\text{edit}(y_{1},y_{2})=\cdots =\text{edit}(y_{t},z)=1\;\leq \;d_{1}$, concluding that f(x)=f(z) with probability of 1. A recent breakthrough was the publication of Order Min Hash (OMH) which was proved to be a gapped LSH (Marçais *et al.* 2019). In order for OMH to guarantee a satisfying hash collision probability, namely sufficiently high for pairs with small edit distance at most $d_{1}$ and adequately low for pairs with large edit distance at least $d_{2}$, the gap between $d_{1}$ and $d_{2}$ is unfortunately quite large, which hinders its practical use. LSH functions for edit distance can also be obtained by first embedding the edit distance into some other metric and then applying a known LSH in the resulting space (Ostrovsky and Rabani 2007; Song *et al.* 2020), but given that embedding for the edit distance often leads to a large distortion, LSH functions designed in this way are also expected to experience a large gap.

**Figure 1.**
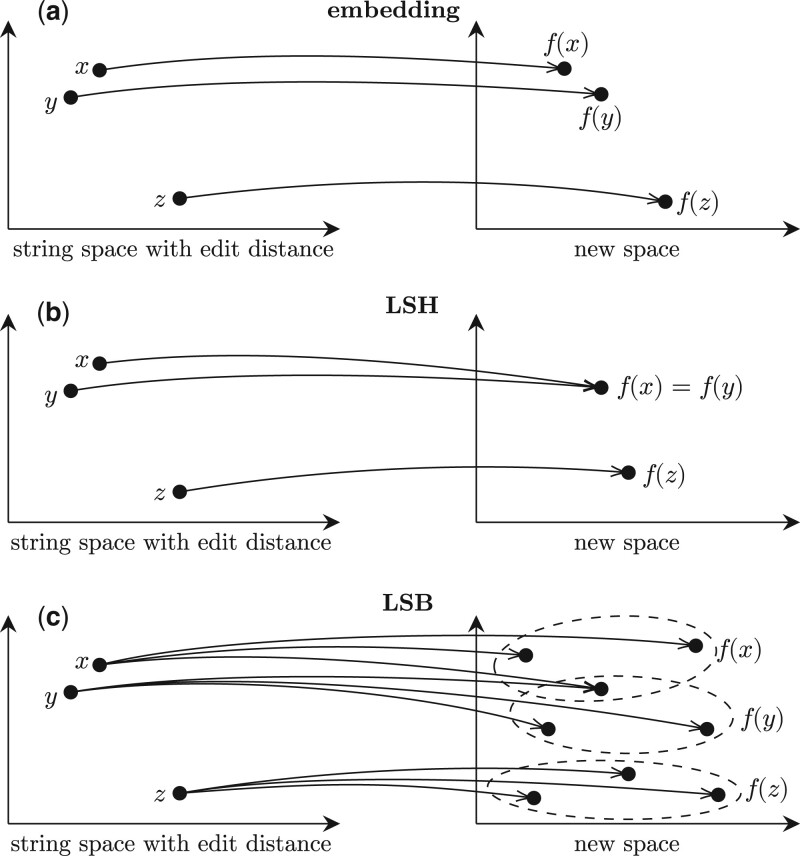
(a) Embedding: similar sequences (in the sense of edit distance) are mapped to similar points (in the sense of a measure of the new space). (b) Locality-sensitive hashing function, which maps similar sequences to the same (hash) value and dissimilar sequences to distinct values. (c) Locality-sensitive bucketing function, which maps similar sequences into overlapping sets and dissimilar sequences into disjoint sets.

We proposed in Chen and Shao (2023) a deterministic generalization of the LSH functions named *locality-sensitive bucketing* (LSB) functions for edit distance that send a sequence to a set of buckets rather than a single one. Such a bucketing function f is defined to be $\left(d_{1},d_{2}\right)$-sensitive if for any two sequences x and y we have that $\text{edit}(x,y)\;\leq \;d_{1}$ implies f(x)∩f(y)≠∅, and that $\text{edit}(x,y)\;\geq \;d_{2}$ implies f(x)∩f(y)=∅. Intuitively, the former condition says that no similar pairs are missed by just comparing sequences within the same bucket, and the latter condition states that dissimilar pairs will not be compared as they will not be assigned into any shared bucket. When requiring that |f(·)|=1, LSB functions become (deterministic) LSH functions. (The embedding, LSH, and LSB functions are illustrated in Fig. 1) In contrast to the nonexistence of any perfect deterministic LSH functions, we demonstrated that gapped and ungapped LSB functions for a variety of $\left(d_{1},d_{2}\right)$ can be constructed, and for a couple of them, including (1,2)- and (1,3)-LSB functions, they are proved to be optimal in the sense of minimizing |f(·)|, i.e., the number of buckets a sequence is sent to.

LSB functions are guaranteed to identify all pairs of sequences with small edit distances while avoiding all excessive comparisons between pairs with large edit distances. This capability makes it scalable for large-scale sequence comparison. However, existing results for LSB functions are limited; designing LSB functions for practical $d_{1}$ and $d_{2}$ with small |f(·)| remains a challenging task. In this work, we seek whether LSB functions can be learned. To this end several challenges need to be addressed. First, it is not clear what is a proper model that can be used to represent an LSB function. Furthermore, unlike learning an embedding function for which a loss function can be written as minimizing the difference between the edit distance and the distance metric of the target space over the training data, i.e., $\sum_{\left(s_{i},t_{i}\right)}|\text{edit}(s_{i},t_{i})\;-\;\mathrm{dist}(f(s_{i}),f(t_{i}))|$, it is unknown what kind of loss functions should be used for learning LSB functions. Here we propose the use of an inception convolutional neural network as the representation, which is built upon minimizer-equivalent units that is able to capture the shared substrings. We also investigate different loss functions and analyze their mathematical intuitions behind. By using a Siamese neural network, the LSB functions can be efficiently trained. We show that for a number of $d_{1}$ and $d_{2}$, the trained LSB functions exhibits nearly perfect accuracy, proving the effectiveness of the training framework. We also compare the learned LSB functions with OMH and demonstrate one application of the learned LSB functions in analyzing erroneous cell barcode data.

## 2 Materials and methods

### 2.1 Problem formulation

**Figure 2.**
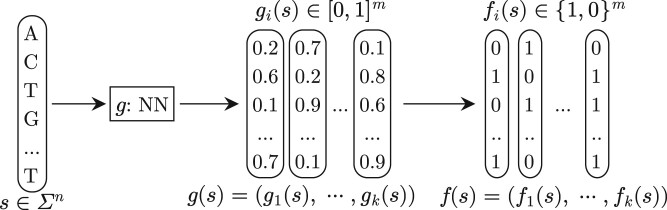
Illustration of the input and output of an LSB function. We use rounded box to represent data and use rectangles to represent components of the neural network throughout the article.

In this work, we represent an LSB function as a neural network followed by a simple rounding procedure. See Fig. 2. The neural network takes a length- n sequence $s\in \Sigma^{n}$ as input, and produces k real-valued vectors, $g(s):=(g_{1}(s),g_{2}(s),\ldots ,g_{k}(s))$, where $g_{i}(s)\in {\left[0,1\right]}^{m}$. Then, all k vectors are rounded to k binary vectors using a threshold θ: elements above θ become 1 and otherwise 0. The resulting k binary vectors are referred to as the k* hash-codes* (i.e., buckets), denoted as $f(s):=(f_{1}(s),f_{2}(s),\ldots ,f_{k}(s))$. The k and m are important parameters of the learned LSB function. We use θ=0.5 throughout this work. (We tested different values of θ and found the accuracy of the resulting LSB-functions is not sensitive to the choice of θ.) We assume Σ={A,C,G,T} in this work but our approach can be applied to any alphabet.

To ease training, we assume that the k hash-codes are “aligned,” i.e., the i-th hash-code of a sequence will only be compared with the i-th hash-code of the other sequence. With this, the definitions of LSB-functions should be slightly modified. We say f that maps a length- n sequence to *k* binary hash-codes to be a $\left(d_{1},d_{2}\right)$-LSB function if it satisfies: for any two sequences $s,t\in \Sigma^{n}$, if $\text{edit}(s,t)\;\leq \;d_{1}$, then, there exists i, 1 ≤ i ≤ k, such that $f_{i}(s)=f_{i}(t)$, and if $\text{edit}(s,t)\;\geq \;d_{2}$, then, $f_{i}(s)\neq f_{i}(t)$ for every 1 ≤ i ≤ k. In this work, we aim to train such aligned LSB functions. When using the trained LSB functions in practice, we also only need to compare aligned hash-codes to determine hash-collision.

### 2.2 Inception convolutional neural network

One key question is the choice of a neural network to represent g. Recall that an LSB function needs to distinguish similar sequences and dissimilar sequences (in terms of the edit distance). One basic observation is that similar sequences more likely share substrings. We believe that the neural network should capture and represent the compositions of substrings of the input sequence. One major challenge of implementing this idea using a machine-learning model is that the shared substrings might be shifted due to the existence of insertions and deletions, and consequently the resulting features will not be “aligned.” In fact, indels is the reason why designing embedding, LSH, and LSB functions for the edit distance is so difficult.

**Figure 3.**
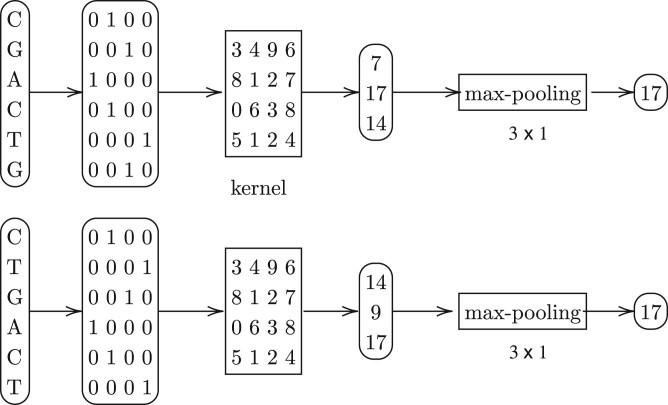
An example of the “building block” the the neural network: the two input sequences (one-hot encoded) have a hash-collision (value of 17, corresponding to the shared 4-mer “GACT”), after going through the *same* convolutional kernel followed by the max-pooling operation.

We propose to use a convolution kernel followed by a max-pooling operation to capture shared substrings. See Fig. 3. As the example shows, this combination is able to handle indels, thanks to the max-pooling operation that selects and therefore “aligns” the features (i.e., shared substrings). It is worth mentioning that such a unit is equivalent to the well-known minimizers, where the convolution kernel acts as a hash-function of the minimizers, and the max-pooling operation exactly does what a minimizer does: picking the minimized/maximized hash-value in the window. This equivalence is revealed and deeply analyzed in Yu (2021).

**Figure 4.**
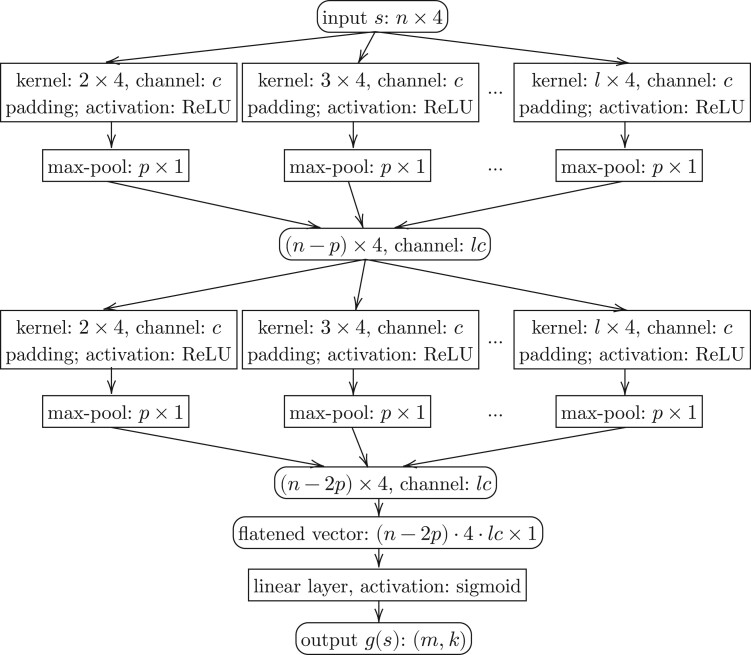
Structure of a two-layer inception convolutional neural network.

The inception convolutional neural network that we use to represent g is depicted in Fig. 4. One inception layer in this model uses multiple above units, to capture substrings of length from 2 to l, where l is a parameter (we use l=9 for n=16 and n=20, and l=13 for n=100). The stride of max-pooling operations ( p in Fig. 4) is another parameter which we find p=2 works better. The output of these units is concatenated and piped into the next inception layer. The number of inception layers can be tuned again according to the sequence length n: in our experiments, we use one inception layer for n=16 and n=20 and two such layers for n=100. The output of the final inception layer will be first flattened and then go through a fully connected linear layer with a sigmoid activation to yield the desired dimension ( m by k).

### 2.3 Training with siamese neural network

**Figure 5.**
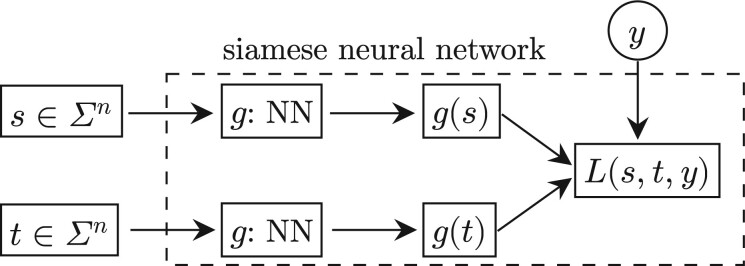
Illustration of a siamese neural network. Note that the two NNs that represent g are identical.

The next key question is how to train an inception convolutional neural network so as to make it satisfying the LSB properties. Unlike ordinary learning tasks of classification or regression which the loss function is defined over a single input/output, the required LSB properties are stated over a pair of inputs/outputs. This distinction makes the training of LSB functions challenging. We identify that the siamese neural network can be used here. Siamese neural network (Chicco 2021) is a class of neural network architectures that contain a pair of identical inner neural networks, which shows preponderance on similarity learning. It has been used in a variety of areas, including image matching (Lee *et al.* 2021), speech emotion recognition (Ntalampiras 2021), sequence embedding, and sequence alignment (Zheng *et al.* 2019). We propose to use the siamese neural network as a training framework in which the inner neural network is above inception convolutional neural network representing g. The two inner models remain synchronized with shared parameters during training, enabling the use of a loss function that involves a pair of inputs/outputs. Figure 5 shows the framework for siamese neural network.

### 2.4 Loss functions

Consider training a $\left(d_{1},d_{2}\right)$-LSB function. Let (s,t,y) be one training sample (see Section “Training Data” for how we generate the data), where $s,t\in \Sigma^{n}$ and y∈{1, − 1} indicating whether $\text{edit}(s,t)\;\leq \;d_{1}$, in which case y= − 1, or whether $\text{edit}(s,t)\;\geq \;d_{2}$, in which case y=1. Let T:={(s,t,y)} be the set of training samples. The entire loss L is simply the addition of the loss over individual samples:

$$L:=\sum_{(s,t,y)\in T}\;L(s,t,y),$$

where L(s,t,y) denotes the loss on sample (s,t,y). We adapt the hinge loss for individual sample:

L(s,t,y):=max{0,1 − 2·y·z(s,t)}.

Note that the inception neural network produces g while the LSB properties are defined using f, i.e., the rounded g. We investigate four different approaches of bridging them, reflected in the forms of z(s,t):

$$\begin{array}{cc} z^{1}(s,t) & :=\overset{k}{\underset{i=1}{\min}}||f_{i}(s)\;-\;f_{i}(t)|{|}_{\infty }\;-\;0.5;\\ z^{2}(s,t) & :=\overset{k}{\underset{i=1}{\min}}||f_{i}(s)\;-\;f_{i}(t)|{|}_{2}\;-\;0.5;\\ z^{3}(s,t) & :=\overset{k}{\underset{i=1}{\min}}||g_{i}(s)\;-\;g_{i}(t)|{|}_{\infty }\;-\;0.5;\\ z^{4}(s,t) & :=\overset{k}{\underset{i=1}{\min}}||g_{i}(s)\;-\;g_{i}(t)|{|}_{2}\;-\;0.5. \end{array}$$

Note that $z^{1}(s,t)$ is the most desirable in the sense of enforcing the LSB properties. To see this, consider a training sample (s,t,y= − 1). If there exists i such that $f_{i}(s)=f_{i}(t)$, then, $z^{1}(s,t)=\;-\;0.5$ and consequently the loss L(s,t,y)=0. This is desired as the trained f satisfies the LSB property on this sample so the loss should be 0. If for every i we have $f_{i}(s)\neq f_{i}(t)$, then, for every i we have $||f_{i}(s)\;-\;f_{i}(t)|{|}_{\infty }=1$ (since $f_{i}$ is a binary vector) and hence $z^{1}(s,t)=0.5$ and consequently L(s,t,y)=2, which is also desired as the trained f does not satisfy the LSB property on this sample, and hence, a positive loss is expected. One can verify similarly that this is the case for samples with y=1.

Other forms $z^{2}$, $z^{3}$, and $z^{4}$ are approximations of $z^{1}$, and they do not enforce the LSB properties as well as $z^{1}$ does. However, they are easier to be trained: the infinity norm is known to be difficult to train comparing with the 2-norm, and the rounding function needed to transform g into f is not differentiable. We compare their accuracies in Fig. 10; in general, using $z^{4}$ yields the highest accuracy.

## 3 Experimental results

In this section, we show the losses in the training process and the accuracies of the trained models. We study different $\left(d_{1},d_{2}\right)$-LSB functions, including ungapped ones where $d_{2}=d_{1}\;+\;1$ and gapped ones. In general ungapped LSB functions are harder to design/train than gapped LSB functions. The choice of parameters k and m are critical (recall that k is the number of hash-codes, and m is the length of each hash-code); we investigate different combinations of k and m. Again, LSB functions become deterministic LSH functions when k=1; we pay attention to the performance for k=1 and k > 1. At the end of this Section we compare the learned LSB with the OMH.

### 3.1 Training data

We only use simulated data to train the models. Given a sequence length n, we randomly generate a large collection of random pairs of length- n sequences {(s,t)} and categorize them by their edit distance, i.e. category- d contains {(s,t)} where edit(s,t)=d. To generate a pair of sequences, we begin by randomly generating a sequence s of length n. Then, a series of d + δ edits are applied on s to obtain a sequence t, where δ is a small integer parameter to offset the possible cancellation of different edits. These edits are sequentially applied, with probability of 1/3 being a subsititution, insertion, or deletion, on a randomly picked position. The resulting sequence t is then padded or truncated to the same length of s. Finally, we compute $d^{\prime }=\text{edit}(s,t)$ and add the pair (s,t) to category-d′.

We study n=20 and n=100, here. For n=20, we use a range of d∈{1,2,…,15}; for n=100, we use a range of d∈{1,2,…,35}. For either n=20 or n=100, we randomly pick 100,000, 20,000, and 50,000 samples from each category in the range, for training, validation, and testing, respectively. To train a specific $\left(d_{1},d_{2}\right)$-LSB function, the edit distance d will be transformed into the label y: samples in category- d will all be labeled as y=−1 if $d\;\leq \;d_{1}$, be labeled as y=1 if $d\;\geq \;d_{2}$, or be discarded if $d_{1}\;<\;d\;<\;d_{2}$ (in the case of training gapped LSB-functions).

All models are trained on an A100 GPU. The training of a one-layer inception neural network for sequences with n=20 takes approximately 4 hours; the training of a two-layer inception neural network for sequences with n=100 takes approximately 23 hours. The testing (i.e., calculating the hash-codes with the trained models) on the test set of 50,000 sequences with n=20 and n=100 takes approximately 162 seconds and 326 seconds, respectively.

### 3.2 Validation loss

**Figure 6.**
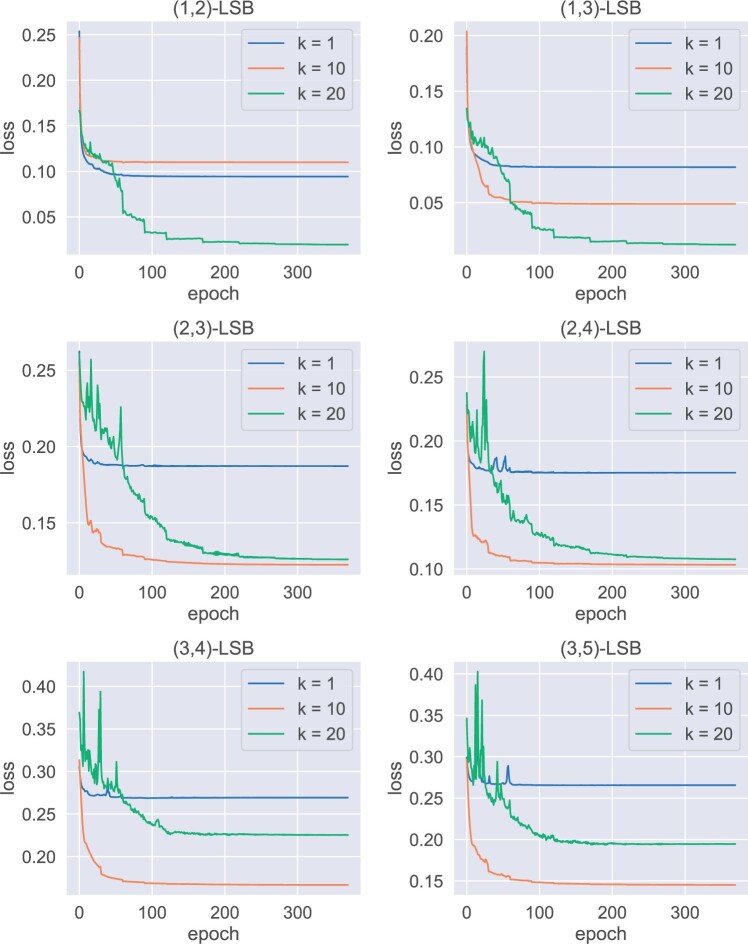
Loss ( y-axis) as a function of epoch ( x-axis); n=20, m=40.

**Figure 7.**
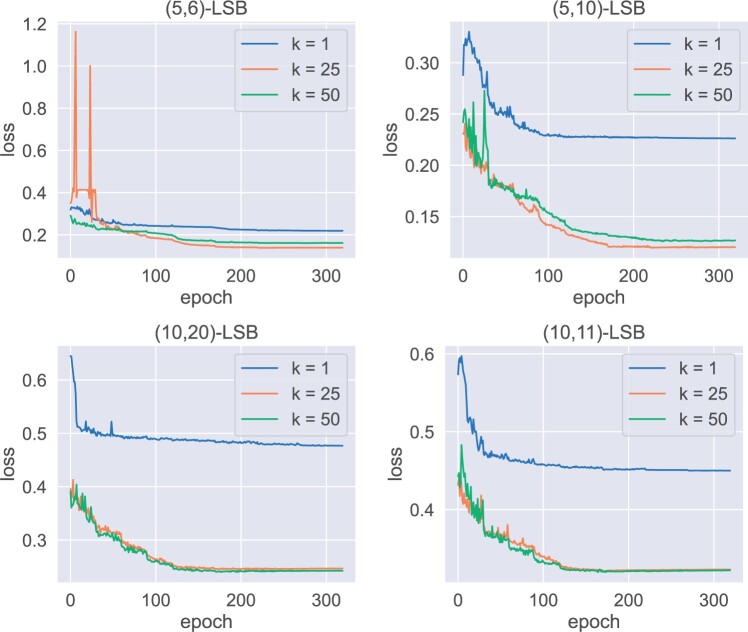
Loss ( y-axis) as a function of epoch ( x-axis); n=100, m=100.

We first investigate the impact of parameter k to the training for different $\left(d_{1},d_{2}\right)$-LSB functions. Figure 6 shows the losses as a function of epoch for short sequence n=20, where m=40. Figure 7 shows the same results but for longer sequences n=100, where m=100. (We also tested other combinations, including n=20,m=20, and n=100,m=50; the resulting patterns are similar.) We observe that in all cases the loss converges, indicating the effectiveness of the training framework. Of interest, one can observe that the loss for k=1 always converges to a high value comparing with larger k, and in many cases the gap is substantial. This shows that it is necessary to use multiple hash-codes to distinguish sequences with small and large edit distances.

### 3.3 Testing accuracy

We then investigate the testing accuracies. Recall that the testing samples are categorized by their edit distance. We first compute the accuracy in each category, termed *categorized accuracy*: let f be the trained $\left(d_{1},d_{2}\right)$-LSB function, a sample (s,t) in category- d, i.e., d=edit(s,t), is said to be predicted correctly if there exists *i* such that $f_{i}(s)=f_{i}(t)$ and $d\;\leq \;d_{1}$, or $f_{i}(s)\neq f_{i}(t)$ for all i and $d\;\geq \;d_{2}$; the accuracy is defined the percentage of correctly predicted instances in the category. The *overall accuracy* is defined as the average accuracy over all categories.

**Figure 8.**
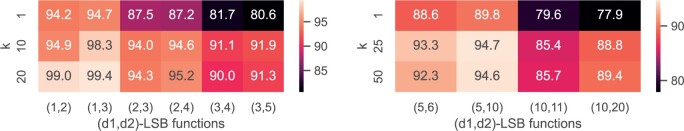
Left: overall accuracy of the learned LSB-functions; n=20, m=40. Right: overall accuracy of the learned LSB-functions; n=m=100.

Figure 8 (Left) show the overall accuracy of the learned LSB-functions for n=20 and various k. Several interesting results can be observed. First, the accuracies of the learned (1,2)-LSB and (1,3)-LSB functions when k=20 are nearly perfect (>99%), which again proves the power of the learning framework that we designed. Second, in Chen and Shao (2023) we proved that an optimal (1, 2)-LSB function (in the sense of minimizing the buckets that a sequence is sent to, i.e., k) requires that k ≥ n=20; this conclusion is also verified: the accuracy of the learned (1,2)-LSB function with a smaller *k*, either k=1 or k=10, is not as close to 1. Third, for “harder” LSB-functions (e.g., $d_{1}\;\geq \;2$) for which we fail to design optimal LSB-functions, using k=1 leads to much reduced accuracy, which again attests the necessity of using multiple hash-codes. Finally, the accuracies of all learned LSB-functions are high (above 0.9 with k=20), suggesting that LSB functions for arbitrary $d_{1}$ and $d_{2}$ can be effectively learned using this framework we established.

Figure 8 (Right) show the overall accuracy for n=100. With larger k such as 25 or 50, we get high accuracy. Note that different from k=25 and k=50, using k=1 will result in much worse accuracy. With k=25 or k=50, the accuracy of gapped LSB functions are higher than that of ungapped functions.

**Figure 9.**
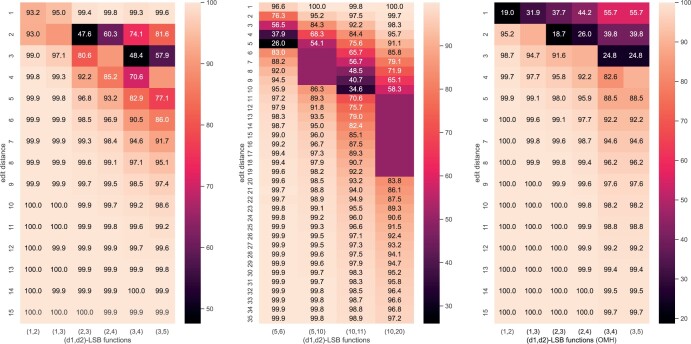
Left: categorized accuracy of learned LSB-function; n=20, m=40, k=20. The gapped edit distances (i.e., larger than $d_{1}$ and smaller than $d_{2}$) are marked blank. Middle: categorized accuracy of learned LSB-function; n=100,m=100,k=50. Right: categorized accuracy of OMH; n=20.

Figure 9 (Left) show the categorized accuracy of the learned LSB functions for n=20 and k=20. From this we can clearly conclude that the “hardest” categories are these around $d_{1}$ or $d_{2}$. This observation suggests future improvement can focus on boundary instances, for example, by using more samples in these categories, or using a Triplet loss, which has been known to be performing better on boundary instances (Dong and Shen 2018). Figure 9 (Middle) show the categorized accuracy for k=50. Again, the bottleneck locates at the boundary edit distances.

### 3.4 Comparison of loss functions

**Figure 10.**
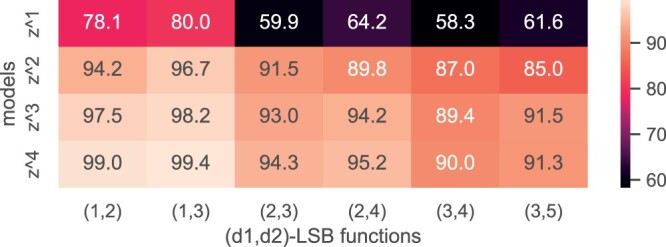
Comparison of the learned LSB-functions for various loss function (n=20, k=20, m=40).

As an investigation of different loss functions described in “Methods,” in Fig. 10 we compare the overall accuracy when different forms of z get used. We can see that $z^{4}$ consistently outperforms; we, hence, use $z^{4}$ as the loss function in all experiments.

### 3.5 Comparison with other neural networks

**Figure 11.**
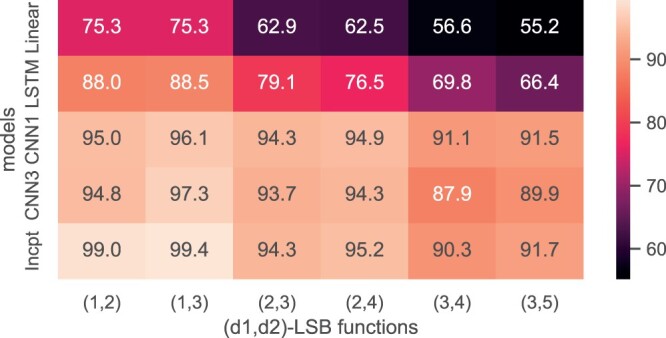
Accuracy of different models; n=20. “Incpt” is our method.

We compare the inception convolutional neural network that we use with several other basic neural networks, including a fully connected linear model, an LSTM model, and two variants of CNN models. The linear model is a four-layer fully connected neural network. The LSTM model contains two layers of LSTM and one linear layer. CNN1 contains four convolution layers, followed by one flatten layer and one linear layer; CNN1 is used to demonstrate the performance of a shallow CNN model. CNN3 is a deeper model that is composed of eight convolution layers, one flatten layer, and one linear layer. Both CNN models also use the same convolution-maxpooling unit as building blocks to capture substrings of length 2 to l. All models are trained and tested with the same set of simulation data. The overall accuracies are given in Fig. 11 for n=20. We can observe that both CNN models significantly outperform linear and LSTM models. This superiority is attributed to the basic units we described previously, which efficiently capture shared substrings. Our inception model further enhanced the CNN models by incorporating uniformly considered substrings within inception layers.

### 3.6 Comparison with Order Min Hash

OMH is the first and the state-of-the-art direct LSH method for the edit distance (Marçais *et al.* 2019). It takes two parameters k and ℓ: given a sequence x of length n, OMH considers its (n − k + 1) kmers and uniquifies each of them by attaching its occurrence number in x. For a random permutation of all such pairs, OMH takes the first ℓ, reorders them according to their positions in x and concatenates the corresponding kmers into a kℓ-mer which serves as the hash code h(x). Two sequences have a hash collision, namely, are in the same bucket, if they produce the same kℓ-mer under the same random permutation. It has been shown that for all ℓ∈[2,n − k] and $0\;<\;d_{1}\;<\;d_{2}\;<\;n$, OMH is $\left(d_{1},d_{2}\right)$-sensitive, i.e., there exists $p_{1}$ and $p_{2}$ such that $\text{edit}(x,y)\;\leq \;d_{1}$ implies $\mathrm{Pr}\left(h(x)=h(y)\right)\;\geq \;p_{1}$ and $\text{edit}(x,y)\;\geq \;d_{2}$ implies $\mathrm{Pr}\left(h(x)\neq h(y)\right)\;\geq \;p_{2}$. In practice, an OMH sketch of a sequence contains m kℓ-mers obtained by repeating with m independent random permutations. In the context of bucketing functions, this is equivalent to assigning each sequence to m buckets, one in each of the m separate groups. The effect is a boosting of the hash collision probabilities: for a pair of sequences x and y, if the probability that they are in the same bucket with one kℓ-mer is p (where the probability is taken over all possible permutations), then, the probability that they share a bucket in at least one of the *m* groups is $1\;-\;{\left(1\;-\;p\right)}^{m}$.

For fixed n, $d_{1}$ and $d_{2}$, it is not clear how to pick the parameters k, ℓ, and m of OMH to achieve the best performance. To compare with the trained LSB functions, we set m to 20 which is the same as the number of hash codes in the trained models; for k and ℓ, a complete grid search for 2 ≤ k ≤ n and 2 ≤ ℓ ≤ n − k is performed and the setting that achieves the best overall accuracy for each $\left(d_{1},d_{2}\right)$ is selected. The parameter choice and overall accuracy are listed in Table 1, where ℓ=2 gives the best result for all $\left(d_{1},d_{2}\right)$ settings. The same set of testing data consisting of pairs of length n=20 sequences is used to evaluate both the trained LSB functions and OMH. The accuracy at each edit distance can be compared at Fig. 9 (Left) and (Right), respectively. It can be observed that the trained LSB functions have significantly higher accuracy than OMH for edit distances below $d_{1}$; in applications, this can lead to much improved sensitivity by capturing a large number of true matches that are potentially missed by OMH. OMH has a slightly better accuracy for edit distances near $d_{2}$. This is largely due to its overall low hash collision probability and the differences vanish soon for larger edit distances.

**Table 1.** Parameters of OMH with the highest overall accuracy.

	$\left(d_{1},d_{2}\right)$					
	(1,2)	(1,3)	(2,3)	(2,4)	(3,4)	(3,5)
*k*	10	6	5	4	3	3
Overall accuracy	94.2	94.5	89.3	89.5	84.4	84.6

The sequence length is n=20, ℓ is 2, and the number of hash codes is m=20.

## 4 Application: barcode assignment

LSB functions are instrumental in comparing error-prone sequencing data. Our training framework is flexible for different applications: users can pick a sequence length n and a suitable $d_{1}$ and $d_{2}$, based on their needs, and train a $\left(d_{1},d_{2}\right)$-LSB function using simulated data. Here we demonstrate its application on handling error-prone cell barcodes. Cell barcodes are nucleotides added to sequencing reads to label the origins of cells. This technology has been widely used in single-cell genomics. Cell barcodes may be erroneous, especially when the error-prone long-reads sequencing technologies are used. Popular pipelines identify correct cell barcodes based on their frequencies (i.e., highly frequent barcodes are more likely to be true), but often discard erroneous barcodes, and consequently discard a significant portion of reads.

We explore the use of LSB functions to “rescue” erroneous barcodes (and their associated reads), i.e., those that do not exactly match any correctly identified barcode (termed whitelist). The idea is that if an erroneous barcode is very close, in the sense of edit distance, to one in the whitelist, then it is likely the origin of the erroneous barcode. Formally, let *B* be the set of the erroneous barcodes, let *W* be the set of barcodes in the whitelist, and let *t* be a threshold of the edit distance, we seek all pairs $P^{*}:=\{(b,w)|b\in B,w\in W,\text{edit}(b,w)\;\leq \;t\}$. A naive algorithm is to perform all-versus-all pairwise comparisons, and for each pair (b,w) determine if edit(b,w) ≤ t. This algorithm requires O(|B|·|W|) number of calculation of edit distance, which is unacceptable as |B| and |W| easily reach the magnitude of millions for modern single-cell datasets. The running time can be reduced to O(|B| + |W|) using (t,t + 1)-LSB function: after generating hash-codes for all barcodes in B∪W which takes O(|B| + |W|) time, one can collect pairs with hash-collisions, i.e., $P_{1}:=\{(b,w)|\exists i\;s.t.\;\;f_{i}(b)=f_{i}(w)\}$. If the (t,t + 1)-LSB function is perfect, the solution will be exact as well (i.e., $P_{1}=P^{*}$); otherwise, the resulting set $P_{1}$ may miss true pairs or contain false-positive pairs. We can use recall, defined as $\left|P_{1}\cap P^{*}|/|P^{*}\right|$, and precision, defined as $\left|P_{1}\cap P^{*}|/|P_{1}\right|$, to measure its accuracy.

There exists another practical, kmer-based algorithm that also runs fast: given a choice of k, reporting pairs that share a kmer, i.e., $P_{2}:=\{(b,w)|S_{k}(b)\cap S_{k}(w)\neq \emptyset \}$, where $S_{k}(\cdot )$ represents the set of kmers in a barcode. In fact, the LSB-based methods and the kmer-based methods can be naturally combined: one can report a pair only if there is an LSB hash-collision *and* they share a kmer, i.e., $P_{3}:=\{(b,w)|\exists i\;s.t.\;f_{i}(b)=f_{i}(w)\;\text{and}\;S_{k}(b)\cap S_{k}(w)\neq \emptyset \}$. It is worth noting that one drawback of kmer-based method and this combined method is that the optimal choice of *k* is unknown in advance.

We test above methods using a dataset from (You *et al.* 2023). The length of cell barcodes is n=16. The whitelist contains |W|=806 barcodes and the erroneous set contains |B|=400,919 barcodes coming from 790,702 reads (multiple reads may have the same erroneous barcode). Note that these reads with erroneous barcodes constitute 27.3% of the total reads (count is 2,899,770) in this dataset.

**Figure 12.**
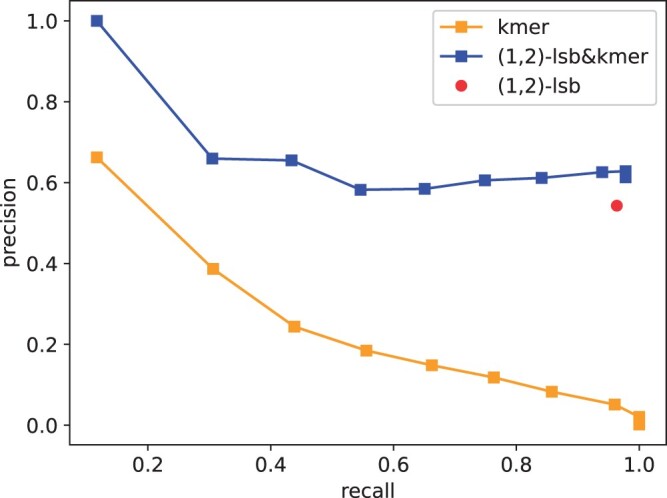
Comparison of accuracy for barcode assignment; t=1. k-mer sizes =5,…,15.

Since the length of the barcode is n=16, it is likely that the erroneous barcodes is only 1 or 2 edits apart from its origins. We therefore consider t=1 and t=2. The accuracies of the above three methods (producing $P_{1}$, $P_{2}$, and $P_{3}$, respectively) when t=1 are given in Fig. 12. One can observe that the LSB-based methods significantly improved the kmer-based method. When kmer is combined with LSB, the accuracy can be further enhanced slightly if a proper k can be picked.

**Figure 13.**
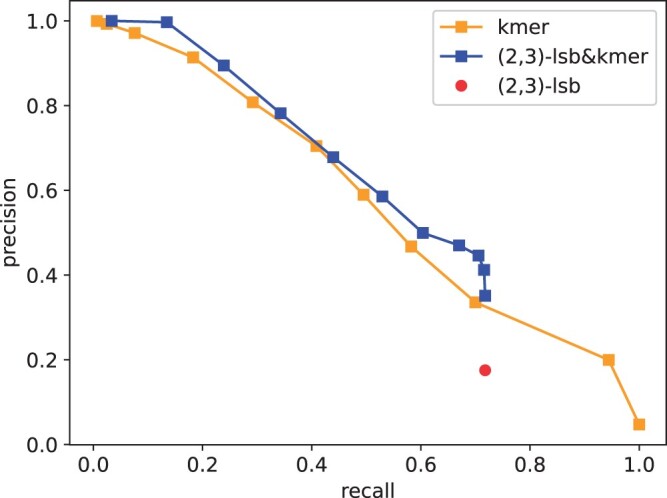
Comparison of accuracy for barcode assignment; t=2. k-mer sizes =5,…,15.

The comparison for t=2 is given in Fig. 13. One can see that kmer-based method outperforms the LSB-based methods. One reason is that the accuracy of the trained (2,3)-LSB is not satisfying as the (1,2)-LSB function. When combined with kmers, the accuracy gets slightly better than kmers-only.

To demonstrate the scalability of our bucketing approach, we compare its running time against the all-versus-all pairwise comparison method using the WFA2-lib (Marco-Sola *et al.* 2023) library. The results are summarized in Table 2. In this application, the sequences are short (n=16) and the edit distance thresholds are small (t=1,2), which are both favorable conditions for the banded wavefront alignment algorithm with early termination. The algorithm is indeed fast, finishing over 323M ( 806×400,919) pairwise edit distance calculations in 1.3x and 1.7x time of the bucketing approach for t=1 and 2, respectively. But in a scaled-up, and hence, more realistic, pairwise comparison scenario where we consider clustering all the erroneous barcodes, although it provides perfect accuracy, the quadratic running time of the all-versus-all approach becomes prohibitively slow. Observe that the bucketing method, having a linear running time with respect to the total number of input sequences, is several orders of magnitude faster.

**Table 2.** Runtime comparison with WFA algorithm (seconds).

	806×400,919		400,919×400,919	
	t=1	t=2	t=1	t=2
WFA	77	104	19 271	26 042
LSB	59	61	71	72

## 5 Discussion

In this work, we establish a learning framework to train arbitrary LSB-functions. We argue that the proposed framework works, evident by the nearly perfect accuracy on a couple of learned functions, high accuracy on several more, and the experimental results that match existing theory. This converts LSB from a promising theoretical concept on paper into a readily accessible tool that many bioinformatics pipelines can benefit from. In particular, any application that wants to tolerate at most *d* edits/errors in the procedure of bulk approximate sequence comparison can utilize a (d,d + 1)-sensitive bucketing function to avoid a majority of the unnecessary pairwise comparisons while enjoying minimal loss of sensitivity.

With this framework, any LSB function with the parameters of users’ choice, including n, k, and m, can be easily trained simply with simulated data. Users can also freely explore different edit distance thresholds $d_{1}$ and $d_{2}$ without the prerequisite of having expertise in designing hashing or bucketing functions. In Supplementary Materials, we show the results of the models trained for n=150, a typical length for NGS data, with reasonable time and memory consumption.

It is very important to boost the accuracy of the LSB functions since it is directly related to the sensitivity and false-positive rates when applied. The accuracy of the learned functions for a variety of $d_{1},d_{2},n$ combinations are suboptimal and we have observed that pairs with an edit distance near $d_{1}$ or $d_{2}$ are more likely to be hashed incorrectly. While it is promising to explore advanced network structures and other loss functions for improvement, we will also investigate sequence-specific network “gadgets” that can capture intrinsic features more directly, for example, network units that considers substrings with a single edit.

As mentioned in the Method section, the trained LSB functions in this work form a variant of the LSB functions defined in Chen and Shao (2023), in the sense that only aligned hash codes are compared. We argue that this variant is not only easier to train (not to be confused with easier to design), but also brings practical benefits such as trivially supporting parallel computation and a reduced number of sequences inside each bucket which can in turn reduce memory usage. Curiously, the optimal (1,2)-sensitive LSB function designed in Chen and Shao (2023) also belongs to this variant and is hence comparable to the one obtained by training. Both the designed and trained functions send a length-*n* sequence into *n* buckets, which has been proved to be necessary for optimality. To better appreciate the trained (1,2)-sensitive function, it is worth noting that the optimality also requires a large bucket universe of size $n|\Sigma {|}^{n\;-\;1}$ which is $5\cdot 2^{40}$ for n=20 and Σ={A,C,G,T}; while the trained function with m=40 only needed a five times smaller universe (i.e., total number of possible hash-codes is $2^{m}$) to achieve its 99% accuracy. Designing such a function by hand would be complicated, if possible at all, further highlighting the strength of our machine learning approach.

The comparison between the trained LSB functions and LSH functions is also interesting and insightful. Technically, an LSH function *f* (such as the ones used in OMH) is uniformly sampled from a finite family of functions F. Consider a (1,2)-sensitive LSH function as a concrete example: Suppose that the first LSH condition says edit(x,y) ≤ 1 implies Pr(f(x)=f(y)) ≥ 0.9. It is interpreted as “for any pair of sequences x and y with edit distance 1, at least 0.9|F| functions in the family maps them to the same bucket.” Now, take a third sequence *z* with edit(y,z)=1 and edit(x,z)=2. A simple cardinality argument shows that at least 0.8|F| functions in the family map x and z to the same bucket. Together with the second half of the LSH property which says edit(x,z) ≥ 2 implies Pr(f(x)≠f(z)) ≥ p, we can conclude that p has to be smaller than 0.2. In other words, a (1,2)-sensitive LSH function that achieves 90% accuracy at edit distance 1 can get at most 20% accuracy at edit distance 2. If repeating is used, namely to sample multiple LSH functions from the family F, to improve the accuracy at 1, then the accuracy at 2 drops further. This dilemma lies in the uniformity of the family of functions and the independence of sampling. The definition of LSB functions, as well as our loss functions, strategically avoid such issue by encouraging diversity and strong correlation among the individual bucketing functions. Using the same example above, if the first hash codes of x and y already produce a collision, the loss function no longer rewards assigning x and y to the same bucket in the remaining hash codes. One can figuratively think that the neural network, as the designer of the bucketing function, is now freed from the constraint that x and y must share a bucket. Being able to take full advantage of such gradually relaxed constraints made it possible to get over 93% accuracy for both edit distances 1 and 2.

Besides handling erroneous cell barcodes demonstrated in the previous section, the trained LSB functions can be useful in many other applications that require large-scale sequence comparisons. For example, the seeding schemes used in read mapping and overlap detection for error-prone long reads can be combined with the LSB functions to improve sensitivity. The LSH methods (such as OMH) used in phylogeny reconstruction and metagenomics binning can be substituted by the LSB functions to boost result accuracy. Our work also extends to various other domains such as predicting homologous protein sequences. Convolutional neural networks have been shown to be effective in protein sequence feature extraction (Yuan *et al.* 2020). Instead of training on edit distance, the model can be easily modified to use the scores of protein sequences as its target for homologous prediction.

A limitation of our current model is that the Siamese network requires the sequences to have the same length. Several workarounds exist in practical use to bypass this limitation. If the input sequences all have similar lengths, they can be padded or truncated as is done in the barcode experiment. The intuition is that a small number of changes at the ends of the sequences has a limited effect on the edit distance (comparing to the possible drastic change on Hamming distance). If the inputs are long and have a large variance in lengths, such as long reads data, we can split each input sequence into one or more parts of equal length with a label pointing to its origin, then perform bucketing on these regular-length pieces. Nonetheless, it is certainly interesting to eliminate this limitation of the model in future work. One possible approach is to preprocess the input sequences into features such that the dimension is united in the feature space before entering the training models.

## Supplementary Material

btae228_Supplementary_Data
