# Supplementary material for: Learning locality-sensitive bucketing functions
Source: Bioinformatics. 2024 Jun 28;40(Suppl 1):i318–27. doi: 10.1093/bioinformatics/btae228 (PMC11211848; doi:10.1093/bioinformatics/btae228)
Supplement: btae228_Supplementary_Data [file btae228_supplementary_data.zip › btae228_Supplementary_Data/Shao.264.sup.1.pdf]

Supplementary Materials for "Learning Locality-Sensitive Bucketing Functions"

Xin Yuan[1], Ke Chen[1], Xiang Li[1], Qian Shi[1], and Mingfu Shao[1,2,*]

[1]Department of Computer Science and Engineering, School of Electronic Engineering and Computer Science, The Pennsylvania State University
[2]Huck Institutes of the Life Sciences, The Pennsylvania State University

March 21, 2024

*Correspondence should be addressed to mxs2589@psu.edu.

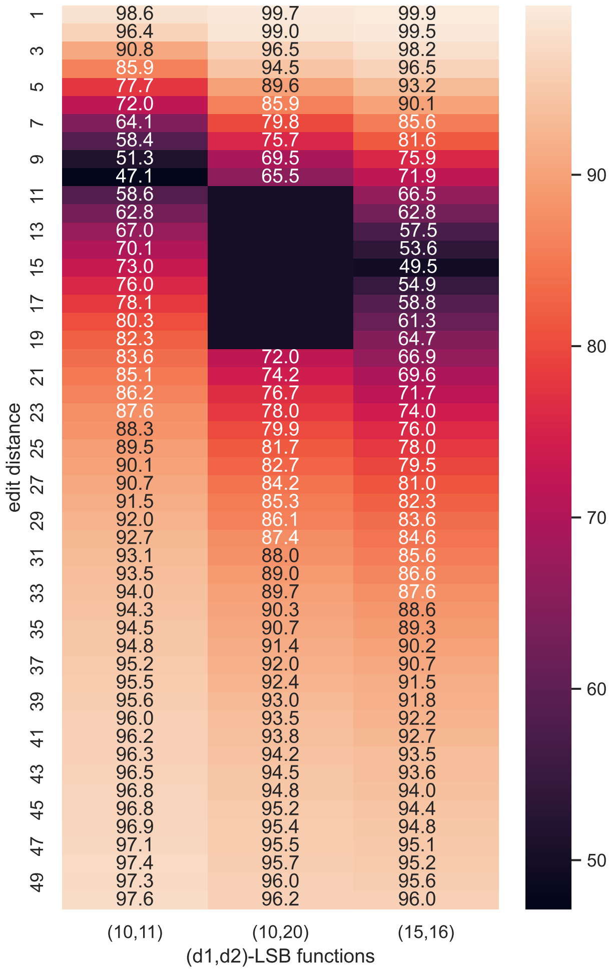

Supplementary Figure 1: Overall accuracy of the learned LSB-functions; $n = m = 150$.
